# Expert Stakeholders’ Perspectives on How Cisgender Heterosexual Boys and Young Men Navigate Sex and Intimacy in Australia: A Case for “Heterosexual Intimacies” in Policy and Practice

**DOI:** 10.1007/s13178-022-00700-3

**Published:** 2022-03-16

**Authors:** Andrea Waling, Alexandra James, Jackson Fairchild

**Affiliations:** The Australian Research Centre in Sex, Health, and Society, La Trobe University, Bundoora, VIC Australia

**Keywords:** Men, Boys, Cisgender, Sexual health, Consent, Dating, Intimacy, Stakeholder, Heterosexual

## Abstract

**Introduction:**

Cisgender heterosexual boys and young men in Australia may experience or perpetuate a range of harms in their romantic and sexual encounters with women due to expectations that they adhere to problematic ideals and norms concerning masculinity and heterosexuality. This paper explores expert stakeholders’ perceptions on these key issues, and their broader implications for policy and practice.

**Methods:**

Using inductive thematic analysis techniques, this paper draws on semi-structured interviews from 23 expert stakeholders working across sectors of gendered violence prevention, sexual health, relationships and sexuality education, sport, and emotional and physical wellbeing.

**Results:**

Findings note several key concerns, including (1) hesitation and lack of adequate information regarding relationships with women; (2) the potential negative influence of pornography; (3) the lack of opportunities to be engaged in sexual health promotion initiatives; and (4) limited opportunities to have meaningful conversations about dating, sex, and intimacy.

**Conclusions:**

Expert stakeholders note several important gaps in policy and practice that need to be addressed to better support cisgender heterosexual boys and young men, and to confront gendered violence and sexual violence.

**Social and Policy Implications:**

Understanding these gaps is vital for policymakers, content and program creators, and service providers working with cisgender heterosexual boys and men. We advocate for thinking about a strategy that is centred around “heterosexual intimacies”, in which addressing boys and young men’s sexual health and wellbeing is brought together with gendered violence prevention and sexual violence prevention initiatives.

## Introduction

Cisgender^1^ heterosexual boys^2^ and young men in Australia may experience a range of harms due to expectations that they adhere to problematic ideals and norms concerning masculinity and heterosexuality (Flood, 2013, 2020). Such norms may include aggression and violence towards others (particularly queer men, queer and straight women, and trans and gender diverse people), hypersexuality, stoicism, and risk-taking behaviours including substance abuse, unsafe sex, and high-risk physical activities (Mahalik et al., 2007; Miller et al., 2016). Other practices include sexually objectifying women, expressing sexual entitlements towards women and trans and gender diverse people (Flood, 2013; Noack-Lundberg et al., 2020; Richardson, 2010), and exhibiting an aggressive heterosexuality that centres/privileges penetration, the phallus, and male pleasure (Beasley, 2015). Due to the burden of having to demonstrate an aggressive heterosexuality, some cisgender heterosexual young men in Australia have reported a lack of recognition of their own refusals in sexual encounters or feeling pressured to perform sexually (e.g., Meenagh, 2021). Indeed, for young cisgender heterosexual men, sex is often discursively configured as an active performance to be judged (Porter et al., 2017). Recent international studies in the USA and UK have also noted an ongoing anxiety and uncertainty among some men when it comes to dating and sex with women (Ford, 2018; Haywood, 2018). These anxieties are amplified by the important gains of the #MeToo movement, which have raised awareness to women’s experiences of sexual violence in dating and other spaces (e.g., Ashwini, 2018). Research notes that on topics like sexual health, expert stakeholders render cisgender heterosexual young men in Australia as invisible or do not regard them as relevant. For example, Connor et al. (2018), in their study of health practitioners working on adolescent pregnancy prevention in the state of Victoria, found that expert stakeholders did not view engaging young cisgender heterosexual men as essential to this work, and instead only focused on young women.

In the wake of increasing acknowledgement of the above issues, there have been calls for Australian legislation to better support gendered and sexual violence prevention programs (Phillips et al., 2015) and mandate sexual consent and healthy relationship education in school-based settings (e.g., Storie, 2021). Australia’s* National Men’s Health Strategy (2020–2030)* (Commonwealth Department of Health, 2019a), for example, includes a focus on ensuring diverse representations of healthy relationships, gender and sexuality in education, inclusion of sexual and reproductive health for heterosexual men, and supporting the modelling of positive role models and behaviours to support the wellbeing of boys and men. *The National Plan to Reduce Violence against Women and their Children 2010–2022* (Council of Australian Governments, 2010) stresses the importance of teaching respectful relationships to young people and supporting positive male attitudes and behaviours. There are several frameworks, programs, and initiatives that run in Australia to respond to the above strategies, including the national comprehensive relationships and sexuality education (RSE) curriculum for school-based programs; masculinity and men’s behavioural change community programs; national gendered and sexual violence prevention programs such as the Respectful Relationships program (Department of Education and Training, 2021); and mental and physical health and wellbeing programs. These all, to varying degrees, are designed to help address some of the noted concerns about young men, sex, sexuality, and sexual intimacy.

In Australia, most formal discussions of RSE happen in school-based settings (Ollis et al., 2021), but this is not consistently applied in schools (or may not be applied at all) (Ezer et al., 2021). Common issues that arise in implementing RSE programs both in Australia and internationally include managing moral, social, and political panics that arise when teaching young people about sex; disagreements concerning the content of RSE curriculum, including whether or not to include discussions of pleasure and intimacy; uncertainty as to who is ultimately responsible for teaching relationships and sexuality education; lack of teacher training; and lack of funds and resources to bring in external experts such as youth workers or sex educators (e.g., Ezer et al., 2021; Pound et al., 2016, 2017). Sexual consent education is also often devoid of considerations of pleasure, with a focus on binary understandings of consensual and non-consensual practices premised within legal and criminological frameworks (e.g., Cameron-Lewis & Allen, 2013; Darnell, 2020; Gilbert, 2018). Respectful Relationships is guided by the national program Our Watch, a recognised leader in the prevention of violence against women and children in Australia. *Change the Story* presents a shared understanding of the evidence and principles of effective prevention and provides a guide to assist governments and other stakeholders to develop their own appropriate policies, strategies, and programs to prevent violence against women (Our Watch, Australia’s National Research Organisation for Women’s Safety (ANROWS) and VicHealth, 2015). While Respectful Relationships teaches about harmful gender role stereotyping to reduce violence towards women, it does not cover any kind of sexual content (Department of Education and Training, 2021). As a result, cisgender heterosexual boys and young men may not have access to adequate information that bridges the link between sexual practices and sexual health; wants, pleasures, and desires; and respectful sexual and intimate relations with women (Cameron-Lewis & Allen, 2013). This gap between sexual practices and desire, pleasure, health, and equitable relationships continues to contribute to broader inequalities.

While there is a plethora of research that critically analyses these programs and initiatives (e.g., Ollis et al., 2021), there is very little research in Australia that explores how expert stakeholders engaged in running or developing these programs or initiatives work with cisgender heterosexual boys and young men on topics concerning sex, sexuality, and intimacy, including challenges that they see cisgender heterosexual boys and young men may be experiencing. Considering how cisgender heterosexual boys and young men may be socialised into problematic engagements in terms of their sexual health and sexual practices (e.g., Flood, 2008), it is prudent to better understand how expert stakeholders might be addressing this in policy and practice. Such stakeholders hold a wealth of knowledge and lengthy experience in working with a large number of cisgender heterosexual boys and young men, including observing how they might be engaging with issues and interventions to address them. In this paper, we seek to explore how expert stakeholders are thinking about men, boys, masculinity, and sexuality when it comes to gender inequalities, sex education, relationships, and intimacy, and gender violence prevention. We draw from a concept of “heterosexual intimacies”, which pays careful attention to how cisgender men and women are socialised into heterosexual relations as part of broader gender socialising. This includes considerations of gendered power dynamics within dating and sexual practices (i.e., men pursue/aggressive, women are pursued/passive), sexual communication expectations (i.e., body language, contraceptive negotiation), and avenues for learning about sex and sexual practices (i.e., pornography) (see Beasley, 2015; Brown, 2015; Crabbe & Flood, 2020; Flood, 2008; Ford, 2018; Haywood, 2018; Meenagh, 2021; Waling, 2022).

We ask, what are the challenges that expert stakeholders see as facing cisgender heterosexual boys and young men when it comes to sex, sexuality, and intimacy? How might topics of sex, sexuality, and intimacy be engaged (or not) when thinking about sexual health and wellbeing, and gendered violence prevention, for this population group? What might be the gaps, implications, or consequences of current strategies, and what can be done to address them? This paper draws from 23 expert stakeholder interviews who work in fields of gendered violence prevention, RSE, sexual health, sport, and mental and physical health and wellbeing across Australia. We explore the key issues that expert stakeholders perceive cisgender heterosexual boys and young men experience when it comes to sex, sexual health, and intimacy.

## Methodology

This study is part of a larger project exploring men’s engagements with sex, consent, and intimacy, which includes interviews and focus groups with young cisgender heterosexual men and women, and cultural analyses of discourses of sex, sexual communication, desires, and gender (Waling, 2022). In this paper, we focus specifically on a subset of this project, that is, engagement with expert stakeholders. We understand expert stakeholders as individuals and organisations who were directly involved in some capacity in working with boys and men on issues of consent; sexuality; intimacy; mental and physical health and wellbeing; and gendered violence prevention. This included conducting workshops in schools or mentoring programs. This also included strategic and policy work, such as developing strategies and initiatives to address issues such as sexual violence towards women.

We consider sexual health to encompass more than just reproductive capabilities and prevention of sexually transmissible infections (STIs) and include a range of components such as sexual consent and gendered violence prevention, mental and physical health and wellbeing, relationships and sexuality education, and pleasure, among others (WHO, 2006). Our rationale in focusing on a range of issues is related to our understanding that sex, consent, and intimacy can be entangled within many facets of one’s life, and may have impacts across varying dimensions of health, relationships, and engagement with or experiences of violence. For example, sexual consent and healthy/respectful relationships may be addressed in RSE programs as well as mental and physical health and wellbeing programs and gendered violence prevention programs. Sexual consent has implications not only for addressing sexual violence, but also for negotiating contraceptive and barrier method use for prevention of pregnancy and STIs and blood-borne viruses (BBVs), and navigating conversations about pleasure, wants, limitations, and desires (Gilbert, 2018). Sporting clubs have also been participating in the gendered violence prevention space to better support gender equality initiatives and address violence towards women, and they include elements of healthy and respectful relationships. Reaching out to a diverse group of expert stakeholders enabled access to a range of viewpoints that could speak to contemporary issues that boys and young men are currently facing in their lives, while also speaking to larger systemic and structural issues in developing and implementing programs to address these issues.

There are limitations to engaging expert stakeholders. Lokot (2021) notes that engaging in key informant interviews (such as those with expert stakeholders) can result in losing the “ordinary” voice of those most deeply affected by these issues. This can include the public and young people. However, the purpose of this study was to better understand expert stakeholder perspectives concerning difficulties cisgender heterosexual boys and young men may be experiencing because of the information with which they are (or are not) engaging, the questions they may be asking, and broader policy and practices that may be shaping these experiences. Expert stakeholders offer a unique insight as they not only work with a range of boys and young men, but also can see and speak to a cross section of social issues. In a subsequent paper, we explore challenges in implementing programs. A separate study as part of the larger project explores young men’s and young women’s experiences of sex, sexual practices, and sexual intimacy.

## Method

Ethics approval for this project was granted by the La Trobe University Human Research Ethics Committee. This paper is focused on this research question: from expert stakeholder perspectives, what are the challenges young men and boys in Australia are facing when it comes to sex, dating, and relationships, and what might be broader systemic or structural issues that contribute to these challenges?

### Data Collection

One-on-one semi-structured interviews and group interviews were conducted with key informant stakeholders working on issues of mental and physical health and wellbeing, programs focusing on masculinity/masculinities, boys and men, domestic and sexual gendered violence prevention, sex education in schools, relationships, sexuality, and sexual health and wellbeing. Purposive sampling was used (Guest et al., 2012). AW curated database of organisations and private consultants across Australia was curated by AJ with support from JF. This included an exhaustive search of organisations that work on one or more of the above key areas. Expert stakeholders were personally invited to take part in the research project from this curated database. Expert stakeholders were sent an email outlining the research study and rationale as to why they were being invited to take part. Eligibility criteria included those expert stakeholders needed to work in a community-based or government program that has some form of engagement with young men and women, either through programs, policy and advocacy work, or social work activities. Expert stakeholders needed to have a focus that included one or more of the following areas: health and wellbeing of young people, RSE, sexual health and wellbeing, gendered violence prevention, and/or boys and men.

For those who expressed interest in participating, one-on-one interviews and small group interviews were conducted using Zoom technology, a video conferencing platform. Data was collected during lockdown restrictions of COVID-19 (between October 2020 and February 2021) thus requiring flexibility in conducting these interviews (Rahman et al., 2021). Participants were therefore offered the choice as to whether they wanted to complete a one-on-one interview or join a group interview. In some cases, group interviews were conducted with a couple of participants from the same organisation. The use of group interviews and single-person interviews were designed to provide complementary forms of data (Lambert & Loiselle, 2008). Participants were invited to participate on a secure, password-protected Zoom link. Zoom was chosen over other platforms such as Skype and Microsoft teams due to its accessibility, and because participants did not require a user account to access this platform. One-on-one interviews were conducted by AW. For group interviews, AWfacilitated the discussions, while AJ kept track of speakers to assist with later transcription.

Participants were asked to talk about the work they do and how they started in that line of work. Questions were then asked of the participants that included exploring: current challenges facing young men in Australia; perceived or experienced barriers in working with men on sex, sexuality, and relationships; current approaches to working with young men, what is working, what is not; and where organisations feel research and understanding should be focused. These topics were used to help facilitate discussion in both group and one-on-one interviews. The open guide format enabled the development of organic conversation and capacity to follow up on new insights emerging from the research. Additionally, in the group interviews, the open guide enabled participants to build off each other to generate further discussion (Plummer, 2017). In this paper, we focus specifically on the first topic: current challenges facing young men in Australia, as understood by expert stakeholders.

Audio recordings were sent to a professional transcriber and then verified by AW. All participants were sent a copy of their transcript to review, a process known as member-checking (Birt et al., 2016). Once transcripts were returned, they were loaded in NVivo for coding and organisation in preparation for analysis.

### Analysis

An exploratory inductive thematic analysis approach was utilised, drawing from Guest et al. (2012). This technique was deemed appropriate as it enabled the research team to see what data emerged in the broader semi-structured group interview and single interview questions pertaining to challenges and issues that expert stakeholders understand cisgender heterosexual boys and young men to experience when navigating sex, intimacy, and dating in Australia. It is also a recommended approach when conducting research that has implications for policy and practice (Guest et al., 2012). To conduct this analysis, the authors first familiarised themselves with the data by reviewing the transcripts and post-interview reflection notes. An iterative codebook using both structural coding and fractured coding techniques (Guest et al., 2012) was then developed after reviewing the data. This was based on these preliminary readings and the semi-structured interview guide, drawing out top-level descriptive codes (key topic or issue raised by the interviewer) and sub-level analytical codes (how interviewees responded to these questions). These key topics/issues included (1) challenges and barriers boys and young men face in Australia; (2) difficulties in working directly with boys and young men; (3) external challenges in working with boys and young men; (4) what broader frameworks, approaches, or intervention strategies are effective; and (5) what broader frameworks, approaches, or intervention strategies are not working or raise criticism. Additional top-level descriptive codes included (1) future research directions as requested by expert stakeholders and (2) demographics of organisations. In this paper, we focus specifically on the top code and underlying sub-codes of (1) challenges and barriers boys and young men face in Australia. AW and AJ cross-checked this codebook, and changes were made accordingly to ensure it accurately reflected the data. The data was then coded by AW and checked by AJ. Analysis was then conducted through reviewing the emergent emic findings, where all three authors met to discuss these through conversations concerning drafts of this paper.

Validity and reliability of the data was done following Guest et al. (2012) recommendations across all three design, collection, and analysis stages. For design, this was achieved through using a whole-team approach through developing drafts of, checking, and finalising the interview guide and questions (design). For data collection, this involved the presence of two interviewers for group interviews and utilising member-checking (Birt et al., 2016), the practice of allowing participants to review their transcripts (collection). For analysis, several recommended data analysis reliability techniques were followed, including developing and using a precise codebook, utilising three external, non-research team member peer review of findings as well as sending research participants a confidential summary report of the findings to provide any feedback or corrections, the creation of an audit trail for coding and analysis, and ensuring the use of supporting quotes from participants in the analysis.

### Participant Demographics

In total, 23 participants in Australia took part in expert stakeholder interviews. Table 1 (above) provides a detailed overview of how we are defining stakeholders.

**Table 1:** Category definitions

**Category**	**Definition**
*Type of expert stakeholder*	
Community	Expert stakeholders are part of organisations that focus on developing communities
Consultant	Expert stakeholders are privately run, often single-person enterprises
Government	Expert stakeholders are part of a government department or program
*Expert stakeholder level*	
Local	Expert stakeholders are focused on supporting local community, so may be restricted to one or more cities or regional centres in providing services, programs, or initiatives
State	Expert stakeholders are focused on services, programs, or initiatives in one state
Multi-state	Expert stakeholders provide services, programs, or initiatives across one or more states
National	Expert stakeholders provide services, programs, or initiatives across Australia (nation-wide)
*Focus of expert stakeholder*	
Boys and men	Programs that are focused on addressing men’s experiences of masculinity or disenfranchisement and supporting young boys and men
Emotional and/or physical health and wellbeing	Programs that are focused on improving emotional and physical wellbeing, including mental health, diet and exercise, and preventing disease
Gendered violence prevention	Programs that are focused on addressing gender inequalities, family, domestic and intimate partner violence, and sexual violence
Sexual health and wellbeing	Programs that focus on relationships and sexuality education in schools, and sexual health promotion, policy, and sexually transmissible infection prevention
Sport and gendered violence prevention	Gendered violence prevention programs that are run through existing sporting clubs

This includes type of expert stakeholder (community, private consultant, government), level of organisation (local, state, multi-state, national), and focus (boys and men, emotional and/or physical health and wellbeing, gendered violence prevention, sexual health and wellbeing, sport and gendered violence prevention). Table 2 outlines demographic characteristics of expert stakeholders.

**Table 2:** Demographic characteristics of participants

Name*	Type of organisation**	Organisation level**	Focus of organisation**	Demographic focus**	**State**	**Interview type**
Alyssa	Community	Multi-state	Boys and men	Young boys and men	NSW, QLD	Single
Andrew	Community	Local	Sport and gendered violence prevention	Young boys and men	VIC	Single
Aubree	Community	National	Gendered violence prevention	Young people (school aged)	National	Group
Bailey	Community	Multi-state	Boys and men	Young boys and men	NSW, QLD	Group
Betty	Community	Multi-state	Sexual health and wellbeing	Young people (school aged)	ACT, NSW, NT, TAS, VIC	Group
Blake	Community	State	Sexual health and wellbeing	Young people (school aged)	VIC	Group
Carly	Community	National	Gendered violence prevention	Young people (school aged)	National	Group
Debra	Community	State	Sexual health and wellbeing	Women	VIC	Single
Declan	Community	Multi-state	Boys and men	Young boys and men	NSW, QLD	Group
Derek	Community	State	Sexual health and wellbeing	Young people (school aged)	VIC	Group
Duke	Community	National	Gendered violence prevention	Young people (school aged)	National	Group
Elsa	Community	State	Sexual health and wellbeing	Young people (school aged)	VIC	Group
Hannah	Government	State	Sexual health and wellbeing	All genders/ages	VIC	Single
Janelle	Community	National	Gendered violence prevention	Young people (school aged)	National	Group
Jason	Community	Local	Sport and gendered violence prevention	Young boys and men	SA	Single
Josie	Community	National	Gendered violence prevention	All genders/ages	National	Group
Julie	Private	Local	Sexual health and wellbeing	Young people (school aged)	VIC	Single
Mahasin	Community	National	Gendered violence prevention	All genders/ages	VIC	Group
Marcia	Private	Local	Gendered violence prevention	Young people (school aged)	VIC	Single
Melissa	Community	National	Gendered violence prevention	Adults	National	Single
Nadia	Government	State	Emotional and/or physical health and wellbeing	Young boys and men	VIC	Group
Patrick	Community	State	Emotional and/or physical health and wellbeing	Young boys and men	ACT	Group
Steven	Community	National	Gendered violence prevention	All genders/ages	National	Group

^*^All names are pseudonyms; ^**^Table 1 provides a breakdown of how these categories are understood in this research paper

Most participants were working in gendered violence prevention spaces (9) or sexual health and wellbeing spaces (7). Over 19 participants worked for a community organisation. These were primarily organisations that functioned at either a national (8) or state-specific level (7). Eleven were based in the state of Victoria, which may be because Victoria has had a significant investment in gendered violence prevention (Department of Premier and Cabinet, 2021) and teaching RSE in schools is mandatory in Victoria (State Government of Victoria, 2010). Most were either working with school-aged (aged under 18) young people (10) or were specifically focused on boys and young men (7). Quotations have been editing for punctuation and clarity.

## Findings

This paper explores the following four challenges: (1) hesitation and lack of adequate information regarding relationships with women; (2) the potential negative influence of pornography; (3) limited sexual health promotion targeting cisgender heterosexual boys and men; and (4) limited opportunities to have meaningful conversations about dating, sex, and intimacy.

### Hesitation and Lack of Adequate Information

Expert stakeholders note a common trend among the boys and men they worked with, that is, an overall hesitation or nervousness as to how to effectively develop sexual and romantic connections with women:

**Derek (Community, sexual health and wellbeing):** I’d say the number one most popular question would be along the lines of ‘What happens if I have sex with someone and then afterwards they take their consent back?’ […] I think that’s what they’re most scared of, they’re absolutely petrified that they’re going to have sexual activity with someone and then get in trouble afterwards.

**Blake (Community, sexual health and wellbeing):** An issue that comes out when speaking to boys is, ‘Well, you know anything I might do might be considered to be inappropriate or might be considered to have crossed the line.’ […] And I think sometimes they want a straight answer to ‘Is this okay or is it not?’, and obviously there’s a lot of grey area and uncertainty around consent and things like that.

Derek and Blake, who run relationships and sexuality education workshops, highlight that while young men are thinking about issues of consent, there is an underlying concern about potential misunderstandings, or doing the wrong thing. Elsa, who also runs relationships and sexuality education workshops, notes concerns about how consent is often approached, highlighting a lack of recognition of men’s experiences of sexual refusal:

**Elsa (Community, sexual health and wellbeing):** They’re the ones that have to get the consent; you know, it’s never that they might be [the ones] saying no.

Here, Elsa notes that boys are positioned in active roles, while women remain in passive roles, highlighting that such approaches can be gender reinforcing rather than gender transformative (Gilbert, 2018).

Carly, who works in the gendered violence prevention sector, notes that there is not enough conversation happening to understand how cisgender heterosexual boys and young men are thinking about or approaching sex, love, and romance:

**Carly (Community, gendered violence prevention):** I think it’s really important to have a conversation with young men about love and what that means to them […] What does a meaningful relationship mean to them now and do they want that, or do they want to have a lot of different sexual partners and just experiment?

For those who did have access to boys and young men’s understandings of sex and relationships, concerns were raised about how problematic ideals and values were generated:

**Bailey (Community, boys and men):** It’s really interesting, because they’re always talking about loyalty, and the woman has to be loyal, and that word comes up all the time […] It seems that they’re really preoccupied with the concept of loyalty, right. And, like, how a woman is meant to show her loyalty to a man.

**Marcia (Private, gendered violence prevention):** The sort of discourse that’s being set for them around sexuality is very male centric, very androcentric in terms of pleasure, and we need to help them break out of that.

Bailey, in running men and masculinity programs, highlights how concepts of “loyalty” become pivotal to boys and young men’s understandings and engagements with romantic and sexual relationships with women, premised around ideas of ownership and subordination. Marcia highlights the need to shift these conversations about sex and relationships for boys and young men.

### Influence of Pornography

The majority of expert stakeholders agreed upon the potential negative influence of pornography on boys and young men’s sexual understandings and relationships with women. This is a longstanding concern that has been researched extensively concerning whether pornography contributes to sexual violence towards women (e.g., Davis et al., 2018; Lim et al.,^2016^), and sexual health difficulties among young men, such as sex addiction (e.g., Duffy et al., 2016) or erectile dysfunction (e.g., Dwulit & Rzymski, 2019). Alyssa, who works in a community program that addresses masculinity, notes the challenges in getting young men to be concerned about their pornography use:

**Alyssa (Community, boys and men):** You know, teenage boys only really get concerned when you say, ‘You know, your dick might stop working.’

Marcia, a leading expert in the influence of pornography use on young people, highlights a few consequences of boys and young men’s potential consumption of pornography:

**Marcia (Community, gendered violence prevention):** Young men are getting a whole range of really problematic messages from pornography – including about gender equality […] Repeated association between porn imagery and experiences of pleasure can create neural pathways, and for most young men, they’re seeing porn literally years before they’ve had a sexual encounter with an actual partner.

Patrick, who provides mentoring programs for young men, reflects on the stories he hears from participants about young men and women’s pornography use:

**Patrick (Community, mental and physical health and wellbeing):** But it’s not only the boys watching the porn, it’s the girls watching the porn that are going, ‘Oh, he wants me to do that’ and then he’s watching it going, ‘Well, she said that; I don’t really want to do [that]’, and then you end up in this space where, as far as I can tell with some of the stories I’ve heard, you’re going ‘Wow, neither of them wanted to do it, but because it was in the movie, that’s what they end up doing,’ and you’re going, ‘How is that possible?’

Patrick highlights how pornography may influence both young men and young women, and that may ultimately shape negative sexual experiences, resting on assumptions of what a partner expects without effective communication.

Others such as Mahasin, who is involved in community gendered violence prevention initiatives, research, and programs, highlight that some young men are aware of the problems of pornography consumption, but lack the skills to address it:

**Mahasin (Community, gendered violence prevention):** Both young men and young women told us that they can see the problems with pornography, but there just isn’t enough education to support them in being able to critically think about that and take that feeling of discomfort further.

In response to this, expert stakeholders like Josie, who works in gendered violence prevention programs, advocate for a pornography literacy:

**Josie (Community, gendered violence prevention):** What we’d really like to see is increased literacy with young people around pornography […] so we want to make sure that young people who are watching porn are seeing some of those things [violence towards women] as they’re occurring, that they can apply some analysis and understanding.

This would, as Josie highlights, assist young men and women in making sense of messaging and images (Crabbe & Flood, 2021).

### Lack of Sexual Health Promotion Focused on Heterosexual Men

Expert stakeholders working in adult sexual health and wellbeing had concerns about the lack of sexual health promotion activities that specifically target cisgender heterosexual young men. For example, Elsa highlights that there has been limited focus on boys and young men’s experiences of body-shaming:

**Elsa (Community, sexual health and wellbeing):** I don’t think men are included in the conversation enough about how these issues [body-shaming] actually impact them as well […] I had a really interesting discussion come up in one of my classes, which is something that I feel really strongly about, is penis shaming, because it’s something that’s actually really big in the media at the moment, and there’s people that are like protesting with signs saying, you know, ‘racism is small dick energy’. And there’s this equation where you equate a small penis size to these horrible values.

Elsa notes a concern around public messaging regarding penis size and penis shaming, highlighting a lack of discussion around this and how it might be shaping boys and young men’s everyday experiences of their bodies. Research has noted that penis size can be a major source of anxiety for men who may not feel they are “large” enough, as there is limited exposure to a diversity of sizes and shapes to support body positivity (Sharp & Oates, 2019).

Debra noted how most sexual health promotion activities for pregnancy prevention are aimed at women:

**Debra (Community, sexual health and wellbeing):** All that sexual reproductive health contraception is targeted at women. The assumption that women are in control of contraception and, yeah, so I can imagine all that leading into the power dynamics we know that happen in male and female heterosexual relationships because of how men and women are raised and what they’re taught […] If a woman did come to the bedroom or a relationship ready to negotiate sex, it being a challenge, if the partner doesn’t know the same.

Debra highlights the impact that contraceptive options have had for women, in which there is an overriding public discourse that positions women as responsible for sexual and reproductive health, with men potentially lacking the necessary skills to navigate those conversations (e.g., Brown, 2015; Wigginton et al., 2018).

A lack of sexual health promotion targeting heterosexual men was echoed by Hannah:

**Hannah (Government, sexual health and wellbeing):** [Discussing men and STI prevention] I think also one of the things that we found, and we found this over a couple of years with the STI testing week campaign, is it’s really hard to have a campaign that resonates with young men […] We haven’t necessarily thought ‘heterosexual males, how do we reach them, support them with targeted messages’ – and we’ve tended to have more of a focus of – we might pick up that heterosexual male if they fall within another priority population group.

Both Debra and Hannah go on to highlight some of the consequences of this, including reproductive coercion, increasing rates of STIs among heterosexual men, and that the long-term serious impacts of STIs (i.e., infertility, cervical cancer) disproportionately impact women:

**Debra (Community, sexual health and wellbeing):** [Discussing impacts of lack of contraception promotion aimed towards men] Coercing a woman into getting pregnant when she doesn’t want to, or coercing her into keeping a baby when she doesn’t want to, or coercing her into having an abortion where she doesn’t want to […] It’s quite complex because that happens, that is a form of violence.

**Hannah (Government, sexual health and wellbeing):** [Discussing men and STI prevention] Hetero men, we’re sitting at an increasing rate [of diagnosed STI infection] than what we have seen in the past […] The majority of the long-term implications of those STI infections are experienced by women not men – the short term is where you might have a rash or a bit of soreness or a discharge or something, but, you know, otherwise it’s no big deal.

Alongside this, Hannah also noted that opportunities for boys and men to be engaged in medical settings concerning sexual health were quite limited:

**Hannah (Government, sexual health and wellbeing):** So, biologically there’s less trigger for a young [heterosexual] male to need to go to a GP [general practitioner] – unless there is, like you said, a cold/flu or they’re off work – they’ve had an injury, they need a medical certificate, or they need an acute intervention for their infection, their injury […] They’re [health clinics] saying, ‘You know, we don’t see the young heterosexual men come into the clinic asking for these tests. You know, they’ve injured themself in some way, or they are coming to our clinic for a particular thing.’ And the health professional then doesn’t say opportunistically, ‘Hey, whilst you’re here, lets [let] you know… are you sexually active, let’s do a sexual health test whilst you’re here’, because the health professional provides a service that that person presented for.

Hannah notes three distinct difficulties. First, as research suggests, cisgender heterosexual men in Australia often avoid engaging in medical health services, particularly sexual health–related services unless they are necessary (e.g., Latreille et al., 2014). Second, health clinics are concerned about bringing up sexual health with young men if it is not the reason the patient is visiting the clinic. This echoes other international studies that have noted that GPs are concerned about discussing sensitive health topics, like sexual health, with young people if young people do not broach the topic first (Jarrett et al., 2011). Third, as young cisgender heterosexual men do not experience menstruation or have need for hormonal contraception, there are less opportunities for them to be engaged in conversations about sexual health in an organic fashion.

### Limited Opportunities to Have Conversations About Sex

Expert stakeholders noted that outside of school-based RSE or Respectful Relationships programs, sexual health promotion initiatives, or clinical settings, there are very limited opportunities for men to be engaged in sexual health and wellbeing discussions, including conversations about consent and relationships. Duke, who works in creating digital content for gendered violence prevention programs, notes that there is no discourse available to young men to begin to have these conversations; this impacts their capacity to develop the skills necessary to begin having discussions about sexual health and related issues:

**Duke (Community, gendered violence prevention):** There’s a whole process of, like, no-one even gives permission for young men to have those conversations with each other. You need that first, and so then you need that to develop the skills, so how do you even do that?

Others such as Melissa, who supports rehabilitation for young men who have engaged in violence, note the lack of opportunity men have had to talk about sexual violence:

**Melissa (Community, gendered violence prevention):** An issue facing men is that there’s not that … it’s not very normalised for men to talk about this [sex and sexual violence] […] A lot of young men say to me when I talk to them about rape culture, or consent, or coercion, or anything like that, they just say, ‘I’ve just never really had a conversation about this in my life. I’ve just never, no-one’s ever talked to me about this, or no-one’s ever asked me, or I’ve never thought about it.’

This is echoed by Carly, who works in policy and practice for gendered violence prevention, highlighting a need to destigmatise this:

**Carly (Community, gendered violence prevention):** It’s [removing the] taboo around men talking about their emotions and having emotions and normalising it, and I think that sort of work is very much needed and can be very powerful.

Genuine interest and curiosity in mutually pleasurable experiences with women was also noted, but there is an absence of conversations supporting men to achieve this, as highlighted by Marcia and Bailey:

**Marcia:** In my experience of working with young men, they often are genuinely interested in something more mutual, and in pleasing their partners. It’s just that it’s not the discourse, no-one is having that conversation with them.

**Bailey (Community, sexual health and wellbeing):** I just don’t, I don’t think that they [young men] know how to pleasure a woman […] [In workshops] they don’t bring it up, actually […] And that would actually be a really good talking point, as to, like, do you have healthy conversations and honest conversations about a woman’s role in sex and how if she’s enjoying it, and are you both getting the same thing out of it?

This is echoed by Blake, who notes a bind in which young men and boys may want to, but feel unable to, to have these conversations:

**Blake (Community, sexual health and wellbeing):** I think, particularly among boys, there’s perceptions of masculinity as well, [which shape] their openness to actually communicate and express their desires. But also, on the other hand, [to have] a desire to ask their partner what they like and what their desires are. And I think it’s all kind of intertwined in them being afraid of doing the wrong thing, but also being afraid to start the conversation and communicate, because that might be perceived as, you know, not such a masculine thing to be doing.

One issue regarding conversations was the lack of focus on, or recognition, of the possibility for boys and young men to have platonic friendships with women, as noted by **Mahasin (Community, gendered violence prevention):**

Often in society we understand male and female relationships as romanticised or sexualised relationships, we don’t necessarily see it as platonic friendships. I’ve seen this happening in primary school, and when teachers talk about friendships they will speak primarily of same-sex friendships and won’t support young people in seeing friendships across different genders.

Derek notes that boys in his workshops are reluctant to discuss issues such as pornography use:

**Derek (Community, sexual health and wellbeing):** Most of the boys that we talk to, if not all of them from primary school onwards, are accessing pornography in some shape or form. But they’re reluctant to talk about it.

Julie, drawing from concerns about approaches to discussing pornography and consent, highlights how such approaches may be hindering efforts to get boys and young men to engage in these conversations:

**Julie (Private, sexual health and wellbeing):** So, all we keep hearing are the demonising of porn, I guess as well, which means that there’s a whole lot of boys looking at porn right now, really worrying about their porn use, and not able to tell anybody […] My other big bugbear at the moment is wanting people to critique consent education, because I think we often, using a legal construct, further demonise those boys that cross the line, and I think they need more guidance than that. We don’t have the expertise to assist them to have a positive conversation about the challenges that they find in being with someone, about the pressure they might feel, or the assumptions they have about being a good man when they’re with someone.

Julie pinpoints two potential consequences in current approaches to handling pornography and consent. First, the demonisation of pornography may in fact make it very challenging for boys and young men to talk about their experiences with others, as Derek notes in his experiences above. Second, the positioning of consent as easy and simplistic may marginalise those who are struggling to fully comprehend and apply the concept.

## Discussion and Conclusion

Discussions with expert stakeholders highlighted several key themes regarding cisgender heterosexual boys and young men. Boys and young men often exhibit uncertainties as to how to engage in mutually respectful and fulfilling sexual, emotional, and romantic relationships with women, particularly as most external messaging presents emotionally fulfilling relationships as forms of controlling ownership. Expert stakeholders also note concerns regarding the consumption of pornographic material by boys and young men without an accompanying conversation that poses a critical perspective on the nature of sex and relationships depicted in pornography. A lack of sexual health promotion or educational initiatives targeting or engaging cisgender heterosexual boys and young men was also noted. The limited opportunities to have conversations about sex, dating, sexual health, and relationships outside of designated RSE school-based programs were also raised.

There are several implications for policy and practice regarding health and respectful relationships. Regarding the challenge of *hesitation and lack of adequate information*, expert stakeholders noted concerning ways in which many boys and young men continue to view women, particularly in terms of one-sided expectations concerning loyalty and respect, and an overall absence of discussion about love, vulnerability, intimacy, and connection. This suggests that more is needed to be done not only to emphasise gender equalities and address harms experienced by women, such as everyday forms of sexual objectification (Gervais & Eagan, 2017), but also to address misconceptions concerning what it means to have a meaningful and mutually reciprocated relationship. Boys and young men’s concepts of loyalty and trust are situated through a lens of control and ownership within romantic relationships, rather than a mutually reciprocated space of comfort and vulnerability. Such ideas can lead to obsessive, coercive, controlling, and violent behaviours in adult romantic and sexual relationships, such as perpetuation of revenge pornography (Henry & Powell, 2018).

Additionally, there is little to no emphasis on the possibility of heterogeneous friendships, with the focus in programs situated on women as *always* a potential romantic or sexual partner. This may in fact contribute to gender inequalities and sexual harms experienced by women. For example, the phenomena of the “friend zone”, whereby men may only view women through a lens of a potential and entitled romantic or sexual encounter. This means men may try to ensure sexual or romantic success through coercive and violent means to avoid the “friend zone” (Marcottee, 2014). Boys and young men’s desire to learn how to give pleasure to women in heterosexual situations has been noted in other studies exploring young people’s perceptions of RSE and sexual health information seeking practices (Litras et al., 2015; Pound et al., 2017). Cisgender heterosexual women continue to report “orgasm gaps” and dissatisfaction with their sexual experiences with cisgender heterosexual men (Mahar et al., 2020). Such inequities within sexual relations are directly linked to the highly criticised androcentric and phallic-centric focus on men’s pleasure in RSE and broader social cultures (Fine & McClelland, 2006). This has important implications for how cisgender heterosexual boys and young men develop effective sexual communication skills (Darnell, 2020; Waling, 2022); critical pornography literacies (Crabbe & Flood, 2021); capacity to negotiate contraceptive and sexual health conversations as well as to take responsibility for their sexual and reproductive health (Brown, 2015); and their capacity to have meaningful discussions with potential partners about sexual wants and desires (Darnell, 2020).

We note that, with most programs designed to be preventative, stakeholders did not comment on how to support boys and young men who may have already engaged in consent violations. This problem is exacerbated by an overall culture of fear created through approaches to sexual consent that are premised within binary yes/no frameworks and situated within legal and criminal framings that lack an adequate consideration of desire and mutual pleasure, and how to manage difficult conversations should something occur (Cameron-Lewis & Allen, 2013; Gilbert, 2018). By this, we mean that in focusing on whether sexual consent is acquired, little room is left to support people in learning how to communicate their sexual wants, needs, and desires. For cisgender heterosexual boys and young men, stakeholders note that this framework positions them as needing to avoid engaging in criminal activity, rather than learning how to engage in effective sexual communication. Boys and young men are concerned about engaging in sex, making it more difficult to then support them in understanding the importance of mutual reciprocation and equality (Darnell, 2020), since focus is premised on whether activities will lead them to potential criminal charges. Here, we note a pressing need to think about how to better support boys and young men who may be reflecting on past incidents, and how to address potential experiences of having committed a violation. As well, the implications of not being able to negotiate sexual experiences leads to grievous harms committed to women who bear the burden of experiencing sexual consent violations and being victim/survivors of sexual violence (e.g., Gervais & Eagan, 2017).

Regarding the second challenge of *the influence of pornography*, concerns were raised about how pornography and sexual consent are being framed and discussed. Such concerns were premised on boys and young men remaining silent regarding personal pornography consumption practices or consent violations. Silences around these can contribute to these being regarded as tabooed topics (Fine & McClelland, 2006). This is important as boys and young men may have limited access to RSE programs that may address these concerns. As some stakeholders note, it is important to create a supportive space to talk about pornography and consent violations, so boys and young men feel more comfortable in discussing their experiences. This also means supporting the development and implementation of critical pornography literacies (Crabbe & Flood, 2021), which include providing young people a set of skills to assess pornography and understand how it may shape their views and expectations concerning sex and pleasure. This would better address Australia’s *National Plan to Reduce Violence against Women and their Children 2010–2022* (Council of Australian Governments, 2010) in supporting young men to learn positive attitudes and behaviours regarding their romantic and sexual engagements with women.

The third challenge, which highlighted *limited focus on cisgender heterosexual boys and young men’s sexual and reproductive health*, as noted by expert stakeholders, is concerning. The *National Update on HIV, Viral Hepatitis and Sexually Transmissible Infections in Australia: 2009–2018* noted a 300% increase (165 to 725 notifications) of reported syphilis infections among women between 2014 and 2019, suggesting increased heterosexual transmission (Kirby Institute, 2019, p. 18). This speaks to expert stakeholders’ concerns about a lack of focus not only on cisgender heterosexual boys and men’s own sexual and reproductive health, but also on how that may inadvertently impact women’s sexual and reproductive health, including their capacity to navigate what can be difficult conversations about contraceptives and barrier-type forms of sexual protection (Brown, 2015). This is not to suggest a shift in focus away from current priority groups who may be at high risk for STIs and BBVs such as HIV in Australia, but rather that we find a way to incorporate cisgender heterosexual men to ensure they take equitable responsibility for their own sexual health and wellbeing. This is important, as such responsibility will also ensure the sexual health and wellbeing safety of their partners. For example, the inclusion of the vaccine for human papillomavirus (HPV) for men in the Australian National Immunisation Program (Commonwealth Department of Health, 2020) in 2013 onwards noted an overall 98% decrease in diagnoses of genital warts among non-Indigenous cisgender heterosexual men, which may support continued reduction in HPV diagnoses (and the potential development of cervical cancer) in people with a cervix such as cisgender women (Kirby Institute, 2019, p. 18). All of these contribute to the last challenge, that is, *the limited opportunity for boys and young men to have meaningful discussions* about sex, sexuality, and intimacy. This can have consequences for the mental health and wellbeing of boys and young men, such as continued reliance on pornography for relationships and sexuality education (Crabbe & Flood, 2021), and anxiety and distress over engaging in sexual intimacy with others.

Australia’s *National Men’s Health Strategy* (2020–2030) (Commonwealth Department of Health, 2019a) now includes a focus on sexual and reproductive health for boys and men. Such focus includes prevention of STIs and BBVs prevention, fatherhood and reproductive health, safe and consensual sexual practices, and testicular and prostate cancer. Australia’s* National Women’s Health Strategy* (2020–2030) (Commonwealth Department of Health, 2019b) has a parallel focus on STI and BBV prevention, motherhood and reproductive health, safe and consensual sexual practices, and cervical and breast cancer, with a greater focus on the high rates of sexual and family violence and its impact than the *National Men’s Health Strategy*. These strategies notably frame men and women’s health issues largely in isolation to each other. For example, issues relating to consent, relationships, and sex are presented in the women’s strategy without specific reference to working with men and boys, and vice versa, with no reference to how relevant initiatives might align across cohorts. Findings in this study thus note a greater need for policymakers and practitioners to conceptualise cisgender heterosexual boys and young men’s sexual health and wellbeing as related to gendered violence prevention initiatives, rather than as entirely separate concepts (Cameron-Lewis & Allen, 2013). This is not to suggest that cisgender heterosexual boys and young men’s sexual practices can only be understood as violent and problematic. Rather, it involves thinking critically about how cisgender heterosexual boys and young men are socialised in which their understanding and enactment of pleasure, desire and sexual activities, and management (or lack thereof) of their sexual health and wellbeing is intricately linked to perpetuating violence towards women (Beasley, 2015; Flood, 2013). It is vital that this is addressed in a wholistic sense.

To do this, we advocate for a consideration of “heterosexual intimacies” in policy and practice across RSE, gendered violence prevention, and mental and physical health and wellbeing sectors. This includes considering more carefully the complex ways in which sex, sexual health and wellbeing, emotional health and wellbeing, and sexual violence are entwined and interconnected in the socialisation of cisgender heterosexual boys and young men. This is noted in research concerning sexual consent (Darnell, 2020; Gilbert, 2018) and the consumption of pornography (Crabbe & Flood, 2021). This includes a greater emphasis and consideration of boys and young men’s responsibility for STI, BBV, and pregnancy prevention; sexual and reproductive health concerns; and emotional wellbeing concerning mutually reciprocal relationships. This means supporting boys and young men to develop the necessary communication skills to achieve this, and paying attention to how gender inequalities emerge within sexual relationships beyond a binary yes/no sexual consent and sexual violence discourse (Darnell, 2020; Gilbert, 2018). This also includes a focus on pleasure, wants, and desire gaps experienced by men and women in sexual situations (Mahar et al., 2020). In other words, we propose taking a wholistic approach that recognises the relationship between boys and young men’s lived experiences of sexual and reproductive health, emotional and mental wellbeing, and potential engagement with violence (and specifically forms of sexual violence) towards women and trans and gender diverse people. At the heart of this discussion is the pressing need to better centre the uncomfortable and difficult conversation about sex, outside of violence prevention and biologically focused sexual health rhetoric, to address gender inequalities and sexual violence more comprehensively and sensitively.

## Data Availability

Due to the nature of this research, participants of this study did not agree for their data to be shared publicly, so supporting data is not available.

## Footnotes

1 Cisgender refers to a person who describes their gender in a way that corresponds to the sex they were assigned at birth.

2 We recognise the cumbersome nature of this phrasing. However, the use of repeated terminology of *cisgender heterosexual boys and young men* is in effort to bring visibility to the diversity of gender and sexuality. We note that people who are sexually or gender diverse are referred to in these ways (i.e., trans women, gay men). In using this language, we attempt to normalise the diversity of gender and sexuality, as opposed to reproducing a dominant paradigm in which ‘men’ and ‘boys’ are presumed cisgender and heterosexual unless otherwise stated.
